# Performances of Clinical, Biological and Echographic Signs to Assess Right Atrial Pressure in Pulmonary Hypertension

**DOI:** 10.3390/jcm15124704

**Published:** 2026-06-17

**Authors:** Magali Croquette, Etienne-Marie Jutant, Elisa Larrieu-Ardilouze, Jean-Eudes Trihan

**Affiliations:** 1 Service de Médecine Vasculaire, Centre Hospitalo-Universitaire de Poitiers, 86000 Poitiers, France; magali.croquette@chu-poitiers.fr; 2 Université de Poitiers, CIC-INSERM 1402, IS-ALIVE Research Group, Centre Hospitalo-Universitaire de Poitiers, Service de Pneumologie, 86000 Poitiers, France; etienne-marie.jutant@chu-poitiers.fr; 3 Service de Cardiologie, Centre Hospitalo-Universitaire de Poitiers, 86000 Poitiers, France; elisa.larrieu-ardilouze@chu-poitiers.fr; 4 Service de Médecine Vasculaire, Centre Hospitalier de Cholet, 49300 Cholet, France

**Keywords:** pulmonary hypertension, acute heart failure, right heart failure, femoral venous stasis index, Doppler ultrasound

## Abstract

**Background/Objectives:** Right heart failure remains the leading cause of death in pulmonary hypertension. Early detection of right-sided congestion is crucial but relies largely on clinical signs with limited diagnostic accuracy. We performed a post hoc analysis of the CODOVEIN study to evaluate the diagnostic performance of clinical and non-invasive parameters for predicting elevated right atrial pressure (RAP). **Methods:** This post hoc analysis included patients from a prospective cross-sectional study who underwent right heart catheterization. Clinical signs, echocardiographic parameters, venous Doppler indices, and NT-proBNP levels were assessed within four hours before catheterization. Patients were stratified according to invasively measured RAP. Diagnostic performances were evaluated using sensitivity, specificity, predictive values, likelihood ratios, and ROC curves. **Results:** Several clinical and imaging parameters were associated with increasing RAP. Among non-invasive markers, the femoral venous stasis index (FVSI) and inferior vena cava (IVC) collapsibility assessed in transverse view showed the best discriminative ability for detecting RAP > 14 mmHg. FVSI demonstrated the highest positive likelihood ratio (>11) and an excellent negative predictive value (>0.98), outperforming other clinical and echocardiographic markers. **Conclusions:** In patients with pulmonary hypertension, FVSI provides robust non-invasive identification of right atrial pressure elevation and may complement traditional clinical assessment for the early detection of right-sided congestion.

## 1. Introduction

Clinical signs of right heart failure have been recognized for centuries. As early as the 4th century BC, Hippocrates described hydrops—characterized by generalized edema, ascites, and dyspnea—distinguishing cardiac, renal, and hepatic origins without understanding the underlying pathophysiology [1]. Despite major advances in cardiovascular medicine, assessment of right-sided congestion remains challenging in clinical practice.

Acute right heart failure (ARHF) manifests through various clinical signs which may be mild or absent, such as peripheral edema, ascites, jugular venous distension, hepatomegaly, and dyspnea [2,3]. However, these signs suffer from limited sensitivity and specificity, particularly in early or slowly progressive congestion [4]. Their interpretation is influenced by interindividual variability, comorbidities, and the tempo of volume overload.

Right heart failure is the primary determinant of prognosis in pulmonary hypertension. Early recognition of elevated right atrial pressure (RAP) is therefore essential for optimizing management and improving outcomes [5]. In routine practice, detection of congestion largely relies on physical examination and indirect biomarkers. However, objective, reproducible and bedside-applicable non-invasive tools to assess systemic venous congestion remain limited.

The CODOVEIN study previously demonstrated that the femoral venous stasis index (FVSI), a Doppler ultrasound index analyzing the percentage of non-anterograde venous flow in the common femoral veins, inspired by the renal venous stasis index (RVSI), predicts elevated RAP and mortality in pulmonary hypertension [6,7]. Building upon these findings, we conducted a post hoc analysis of CODOVEIN to compare the diagnostic performance of multiple clinical and non-invasive parameters for identifying significant RAP elevation.

## 2. Materials and Methods

This work represents a post hoc analysis of the CODOVEIN study, which aimed to evaluate the diagnostic performance of the femoral vein Doppler index (FVSI) for identifying elevated right atrial pressure in patients with pulmonary hypertension. The original study was designed as a prospective cross-sectional investigation conducted between 1 July 2021 and 30 November 2022 at a tertiary care center in Poitiers, France, within a dedicated pulmonary hypertension referral unit. Consecutive adult patients admitted for suspected pulmonary hypertension or routine follow-up and undergoing right heart catheterization (RHC) were included.

For each participant, a comprehensive assessment was performed, including clinical examination, echocardiography, femoral venous Doppler ultrasound, and laboratory analyses. All non-invasive measurements were obtained within four hours prior to RHC, with the exception of NT-proBNP, which could be collected up to 48 h before inclusion. No diuretic therapy was administered within the three hours preceding inclusion or during the RHC procedure. The full study protocol has been published previously [6].

The present post hoc analysis was specified prior to data extraction and focused on clinically meaningful non-invasive indicators of congestion. The study received approval from a national ethics committee (2021-A00100-41) and was registered on ClinicalTrials.gov (NCT04792879).

### 2.1. Clinical Signs

Lower limb pitting edema was defined as ankle swelling confirmed by the patient associated with pitting. For hepatojugular reflux, abdominal pressure was applied at the upper right abdominal quadrant for approximately 10 s. Sustained clinical distension of jugular veins was considered a positive hepatojugular reflux. Jugular venous distension was defined as a spontaneous distension of the jugular veins, in a patient placed in a semi recumbent position (approximately 30°). Last, clinical ascites was defined by an increase in abdominal circumference associated with at least one of the following signs: dullness (detected by abdominal percussion), rigidity of the abdominal wall or flattening of the umbilicus.

### 2.2. Echographic Measurements

All examinations were performed by two experienced vascular physicians who were blinded to right heart catheterization results at the time of ultrasound acquisition. Inter-operator reproducibility of venous Doppler measurements was evaluated in the present study and has been reported previously [6].

For FVSI assessment, both common femoral veins were examined using Doppler ultrasound. Patients were positioned in a strict supine position, with a maximum upper body elevation of 15° when required due to dyspnea, and rested for at least 5 min prior to measurements. Heart rate was continuously monitored using electrocardiography. Ultrasound imaging was performed with a 9 MHz linear transducer (GE Vivid E95; GE Healthcare Systems, Chicago, IL, USA) using standard coupling gel.

Venous flow in the common femoral vein was recorded with the probe placed just below the inguinal ligament, approximately 1–2 cm above the femoral head. Doppler signals were obtained from a longitudinal view, with color Doppler box size adapted to vessel diameter. The ultrasound beam was aligned as parallel as possible to the direction of flow, with an insonation angle ≤ 60°, and the sample volume positioned at the center of the vein.

For each acquisition, patients were instructed to briefly hold their breath at the end of a normal inspiration (approximately 5 s), allowing a Doppler recording of about 3 s. FVSI (unitless) was calculated as: (cardiac cycle duration [ms] − anterograde flow duration [ms])/cardiac cycle duration [ms], in accordance with previous reports [6]. In the absence of cyclic retrograde flow, FVSI was set to 0. Measurements were performed bilaterally, and the highest value was retained for analysis.

RVSI measurements were subsequently obtained with the patient in a lateral decubitus position. The renal veins were assessed at the hilum using a low-frequency convex transducer (1.5–6 MHz). RVSI was calculated as: (cardiac cycle duration [ms] − anterograde venous flow duration [ms])/cardiac cycle duration [ms] [7,8]. To minimize respiratory and intra-abdominal pressure variations, patients were asked to hold their breath at the end of a quiet inspiration. Both renal veins were evaluated, and the highest value was retained. As for FVSI, RVSI was set to 0 when no cyclic retrograde flow was observed.

For longitudinal IVC assessment, the maximal IVC diameter was measured along the long axis during end-expiration, 1–3 cm proximal to the right atrium, using a subcostal view with a phased-array probe (1.5–4.6 MHz), during both normal respiration and inspiratory sniff.

For transverse IVC measurements, the minimal (short-axis) diameter was obtained at end-expiration approximately 1 cm above the hepatic veins using a retrohepatic approach with a convex probe (2–9 MHz), again during normal breathing and inspiratory sniff.

The IVC collapsibility index was defined as the ratio between the IVC diameter during inspiratory sniff and that measured during normal respiration.

### 2.3. Right Heart Catheterization

Right heart catheterization (RHC) was performed in accordance with current guideline recommendations [8]. Pressures were recorded at the level of the right atrium (RA), right ventricle (RV), and pulmonary artery. Cardiac output (CO) was determined using the thermodilution method.

Hemodynamic parameters were retrieved from electronic medical records, including systolic pulmonary artery pressure (sPAP), mean pulmonary artery pressure (mPAP), mean right atrial pressure (RAP), pulmonary artery wedge pressure (PAWP), CI, pulmonary vascular resistance (PVR), and mixed venous oxygen saturation (SvO_2_). PVR was calculated as (mPAP − PAWP)/CO.

For the primary endpoint, RAP values were stratified into three categories (“<8 mmHg”, “8–14 mmHg”, and “>14 mmHg”) based on pulmonary hypertension management guidelines [8].

### 2.4. Statistical Analysis

Patients were classified according to invasively measured RAP into predefined categories (<8, 8–14 and >14 mmHg) according to PH management and follow-up guidelines. Continuous variables were analyzed using one-way ANOVA or Kruskal–Wallis test as appropriate. Categorical variables were compared using chi-square test or ANOVA depending on group number. All tests were two-sided, and a *p*-value < 0.05 was considered statistically significant.

Diagnostic performance was assessed by calculating sensitivity, specificity, positive and negative predictive values, positive and negative likelihood ratios, and area under the ROC curve. Optimal thresholds for quantitative variables were determined using ROC analysis to maximize sensitivity and specificity balance.

## 3. Results

One hundred and one patients were included in the analysis, with 91 patients with confirmed PH on RHC. Baseline characteristics were stratified according to invasively measured right atrial pressure (RAP) are presented in Table 1. Estimation of FVSI, RVSI, longitudinal and transversal IVC collapsibility was inaccessible in 4 (4%), 29 (28.7%), 11 (10.9%) and 20 (19.8%) patients, respectively.

Table 2 summarizes diagnostic performances of the studied variables. Elevated RAP was associated with a higher prevalence of clinical signs of systemic congestion, including peripheral edema, jugular venous distension, and ascites. Echocardiographic parameters reflecting right-sided filling pressures, such as right atrial area and inferior vena cava (IVC) collapsibility, deteriorated progressively across RAP categories. NT-proBNP concentrations increased with RAP but showed substantial overlap between strata.

Venous DUS indices demonstrated a strong relationship with RAP severity. Both the femoral venous stasis index (FVSI) and renal venous stasis index (RVSI) increased significantly with higher RAP, with FVSI showing the most pronounced gradient across pressure categories. This finding supports a close relationship between peripheral venous flow abnormalities and systemic venous congestion.

For the detection of severe RAP elevation (>14 mmHg), FVSI and IVC collapsibility assessed in the transverse (retrohepatic) view provided the best discriminative performance. FVSI achieved an area under the ROC curve of 0.93, with a positive likelihood ratio exceeding 11 and a negative predictive value above 0.98. Transverse IVC collapsibility also demonstrated good diagnostic accuracy, with a positive likelihood ratio greater than 4.5.

In contrast, longitudinal IVC collapsibility and NT-proBNP showed high negative predictive values (>0.90) but limited discriminative ability, with positive likelihood ratios below 2. Clinical signs such as peripheral edema, jugular venous distension, and hepatojugular reflux exhibited modest sensitivity but good specificity. Ascites showed very high specificity but no calculable sensitivity or positive predictive value due to the absence of true-positive cases to detect a RAP > 14mmHg.

## 4. Discussion

Accurate assessment of volume status and right-sided congestion has challenged clinicians for millennia. Physical examination findings depend on patient-specific factors and the rate of congestion development, limiting their diagnostic reliability. Our findings confirm the modest performance of traditional clinical signs for detecting elevated RAP but outline the diagnostic value of FVSI and IVC collapsibility if measured on a transverse section.

Notably, clinical signs of right-sided heart failure, including jugular venous distension, hepatojugular reflux, and ascites, demonstrated high specificity and positive predictive value for elevated right atrial pressure. This suggests that their presence may reliably support the diagnosis of increased RAP. However, their absence does not exclude elevated RAP, highlighting their limited sensitivity and reinforcing the need for complementary non-invasive tools. Ultrasound-based evaluation of the inferior vena cava has gained widespread use, although randomized evidence remains inconclusive. In particular, Jobs et al. did not demonstrate improved outcomes with IVC-guided decongestive therapy in acute decompensated heart failure [9], underscoring the need for complementary tools.

In our study, IVC collapsibility showed better diagnostic performance when measured in a transverse (retrohepatic) view compared with the conventional longitudinal (substernal) view. This difference may be explained by the physiological motion of the IVC during inspiration. As the diaphragm descends and flattens, the IVC shifts anteriorly and laterally, making it difficult to continuously track its maximal diameter in a longitudinal view. In contrast, the retrohepatic transverse approach aligns the ultrasound beam more closely with this displacement, allowing a more stable assessment.

This hypothesis is supported by the systematically lower diagnostic thresholds observed with longitudinal measurements compared to transverse ones (48% vs. 58% for RAP < 8 mmHg, and 39% vs. 47% for RAP > 14 mmHg, respectively). In a longitudinal view, failure to maintain alignment with the true maximal diameter may lead to an overestimation of collapsibility at a given RAP.

In contrast to IVC assessment, which is highly dependent on respiratory effort, body position, and probe alignment, Doppler evaluation of the common femoral vein reflects downstream venous congestion and appears less susceptible to these limitations. This may explain the superior diagnostic performance of FVSI observed in our study. In our cohort, FVSI was measurable in more than 96% of patients, compared to 71–89% for other ultrasound indices, supporting its robustness and feasibility at the bedside.

In the present analysis, TAPSE and right atrial (RA) area showed weaker discrimination across RAP strata compared with other echocardiographic parameters, with only RA area reaching statistical significance and a modest gradient between groups. This relatively limited performance may be explained by their intrinsic physiological characteristics. TAPSE primarily reflects longitudinal right ventricular (RV) function and may therefore not adequately capture global RV performance, particularly in the context of pressure overload such as pulmonary hypertension, where radial and septal components also contribute significantly. Similarly, RA area reflects structural remodeling and chronic adaptation to pressure and volume overload rather than acute hemodynamic changes, which may limit its sensitivity to detect variations in right atrial pressure. These findings suggest that, although widely used in routine practice, TAPSE and RA area may be less suitable for the assessment of RAP in this specific clinical setting compared with more dynamic or flow-based indices.

An important aspect to consider when interpreting our findings is the differential feasibility of the echocardiographic parameters evaluated. RVSI and transversal IVC measurements were more frequently limited by suboptimal acoustic windows or technical constraints inherent to Doppler acquisition, resulting in a higher proportion of missing data. These limitations likely reflect the dependence of these indices on adequate alignment, image quality, and operator expertise. In comparison, other parameters appeared more consistently obtainable across patients, although feasibility was not formally quantified. Importantly, such variability in acquisition conditions may have influenced the observed associations with RAP and should be taken into account when interpreting comparative performance. More broadly, these findings underscore that beyond intrinsic diagnostic properties, the practical feasibility of echocardiographic indices represents a key component of their clinical applicability, particularly in patients with pulmonary hypertension, in whom imaging conditions are often challenging. In this context, recent advances in imaging modalities suggest that more comprehensive approaches, such as three- or four-dimensional echocardiography, may help overcome some of the geometric and alignment limitations inherent to conventional two-dimensional techniques, particularly for complex right heart and tricuspid valve assessment [10].

In the STRONG-HF trial, Mebazaa et al. demonstrated that optimization of guideline-directed medical therapy combined with close follow-up significantly reduced morbidity and mortality [11]. Even if this notion seems obvious in medical practice, this study has shown the importance of rapid up-titration of guideline-recommended therapies: Angiotensin converting enzyme inhibitors, Angiotensin II receptor blocker, or Angiotensin Receptor–Neprilysin Inhibitor and especially β blockers. However, in their study protocol, congestion assessment relied solely on physical examination and NT-proBNP, without specific imaging guidance recommendations, thus highlighting the unmet need for objective non-invasive markers of congestion to safely guide treatment intensification.

Recent advances such as the natriuretic response prediction equation (NPRE) allow good prediction of diuretic response but do not directly assess venous congestion [12,13,14]. Venous Doppler indices, particularly FVSI, directly reflect systemic venous hemodynamics and could thus be a strong complementary tool to NPRE.

Our results suggest that FVSI provides robust and reproducible identification of severe right-sided congestion and may enhance clinical decision-making when integrated with standard assessment. Beyond the detection of congestion, FVSI could also represent a simple and accessible tool for risk evaluation in patients with pulmonary hypertension. Indeed, contemporary management strategies increasingly rely on multiparametric risk stratification models integrating clinical status, exercise capacity, biomarkers, echocardiographic findings and invasive hemodynamics to guide therapeutic decisions and treatment escalation, as emphasized in recent guidelines [8]. Within these frameworks, right atrial pressure and markers of right heart dysfunction are recognized as important prognostic determinants, and follow-up hemodynamic variables have been shown to strongly predict outcomes in pulmonary hypertension [5,15]. In this context, a non-invasive surrogate of elevated RAP such as FVSI could contribute to refining bedside risk evaluation and longitudinal monitoring. By providing a rapid and reproducible assessment of systemic venous congestion, FVSI may therefore complement established risk stratification tools and potentially support earlier therapeutic adjustments in routine clinical practice.

### Limitations

This post hoc analysis is derived from a pulmonary hypertension population, which differs from the broader heart failure population; therefore, generalization to other clinical settings should be made with caution. However, a substantial proportion of patients in our cohort presented with either isolated post-capillary pulmonary hypertension or combined pre- and post-capillary pulmonary hypertension, which may support a certain degree of transposability to heart failure populations with elevated left-sided filling pressures, although this remains to be formally demonstrated.

Although the analysis was pre-specified, the post hoc design inherently limits causal inference and warrants prospective validation in larger and more diverse cohorts. In addition, the relatively small number of patients with markedly elevated right atrial pressure (RAP > 14 mmHg) may limit the robustness of diagnostic performance estimates in this subgroup, and these findings should be considered hypothesis-generating. The absence of correction for multiple comparisons may also increase the risk of type I error. Finally, missing measurements for certain Doppler parameters, particularly RVSI and transversal IVC indices, may have introduced selection bias and should be acknowledged when interpreting the results.

## 5. Conclusions

Femoral venous Doppler assessment using the FVSI offers superior non-invasive detection of elevated right atrial pressure compared with traditional clinical and echocardiographic markers in patients with pulmonary hypertension. FVSI may represent a valuable adjunct to existing non-invasive tools for the assessment and management of right-sided congestion, particularly in patients with pulmonary hypertension.

## Figures and Tables

**Table 1:** Baseline characteristics stratified by right atrial pressure.

Variables	All Patients (n = 101)	RAP < 8 mmHg (n = 47)	RAP 8–14 mmHg (n = 43)	RAP > 14 mmHg (n = 11)	*p*-Value *
**Demographics**					
Age (years old)	70 [63, 74]	70.0 [63.5, 74.5]	70.0 [62.0, 73.0]	75.0 [66.5, 76.5]	0.413
Sex					
Female (%)	49 (48.5)	20 (42.6)	26 (60.5)	6 (54.5)	0.231
Male (%)	52 (51.5)	27 (57.4)	17 (39.5)	5 (45.5)	
**PH hemodynamic classification**					
Absence of PH (%)	10 (9.9)	9 (19.1)	1 (2.3)	0 (0.0)	<0.001
Pre-capillary PH (%)	56 (55.4)	32 (68.1)	22 (51.2)	2 (18.2)	
Combined post and pre-capillary PH (%)	24 (23.8)	3 (6.4)	17 (39.5)	4 (36.4)	
Isolated post capillary PH (%)	11 (10.9)	3 (6.4)	3 (7.0)	5 (45.5)	
Unclassified	1 (1.1)	1 (2.6)	0 (0.0)	0 (0.0)	
**Clinical signs of right heart dysfunction**					
Lower limb pitting edema (%)	27 (26.7)	6 (12.8)	15 (34.9)	6 (54.5)	0.06
Hepato-jugular reflux (%)	26 (25.7)	4 (8.5)	15 (34.9)	7 (63.6)	<0.001
Jugular venous distension (%)	14 (13.9)	2 (4.3)	9 (20.9)	3 (27.3)	0.029
Clinical ascites (%)	3.0 (3.0)	0 (0.0)	3 (7.0)	0 (0.0)	0.124
**Biological data**					
NT-pro-BNP (ng/L)	354 [122, 1969]	166 [82, 344]	568 [342, 3471]	1221 [438, 1920]	<0.001
**Echocardiography**					
TAPSE	19 [16, 23]	20 [17, 23]	18.5 [15.75, 22]	18.5 [10.75, 22,5]	0.486
RA area	21 [16.75, 25]	18 [15, 24]	23 [18.5, 25]	24.5 [20.75, 26.5]	0.048
IVC-estimated RAP					
0–5 mmHg (%)	43 (50)	23 (59.0)	17 (44.7)	3 (33.3)	<0.001
6–10 mmHg (%)	29 (33.7)	14 (35.9)	15 (39.5)	0 (0.0)	
11–20 mmHg (%)	14 (16.3)	2 (5.1)	6 (15.8)	6 (66.7)	
**IVC collapsibility**					
Longitudinal IVC collapsibility	0.43 [0.26, 0.60]	0.55 [0.38, 0.64]	0.37 [0.24, 0.59]	0.19 [0.12, 0.42]	0.006
Longitudinal view: IVC inaccessible (n (%))	11 (10.9)	-	-	-	-
Transversal IVC collapsibility	0.68 [0.46, 0.82]	0.77 [0.64, 0.83]	0.67 [0.46, 0.79]	0.32 [0.28, 0.42]	< 0.001
Transversal view: IVC inaccessible (n (%))	20 (19.8)	-	-	-	-
**Doppler ultrasonography**					
Femoral venous stasis index	0.24 [0.0, 0.34]	0.0 [0.0, 0.17]	0.30 [0.22, 0.37]	0.49 [0.46, 0.54]	<0.001
FVSI inaccessible (n (%))	4 (4)	-	-	-	-
Renal venous stasis index	0.12 [0.0, 0.19]	0.0 [0.0, 0.15]	0.13 [0.08, 0.31]	0.24 [0.10, 0.30]	0.002
RVSI inaccessible (n (%))	29 (28.7)	-	-	-	-

* After application of the Bonferroni correction, *p* < 0.004 should be considered significant. Values are expressed as median [interquartile range] unless otherwise stated. Comparisons were performed across RAP categories using appropriate statistical tests: one-way ANOVA or Kruskal–Wallis test and khi-2 or Fisher test depending on statistical prerequisites. IVC: Inferior vena cava. NT-pro-BNP: N-terminal pro-brain natriuretic pressure. PH: Pulmonary hypertension. RA: Right atrium. RAP: Right atrial pressure. TAPSE: tricuspid annular plane systolic excursion.

**Table 2:** Diagnostic performances of clinical, echocardiographic and echographic signs to detect right atrial pressure <8 mmHg or >14 mmHg.

Variables	Cut-Off Values	AUC	Sensitivity	Specificity	PPV	NPV	PLR	NLR
**To detect an RAP > 8 mmHg**								
RVSI	0	0.73 [0.62–0.85]	0.83 [0.67–0.94]	0.61 [0.43–0.77]	0.68 [0.52–0.81]	0.79 [0.59–0.92]	2.14 [1.39–3.31]	0.27 [0.13–0.59]
FVSI	>0.19	0.88 [0.81–0.95]	0.87 [0.75–0.95]	0.81 [0.67–0.92]	0.85 [0.73–0.94]	0.83 [0.69–0.93]	4.68 [2.48–8.82]	0.16 [0.08–0.32]
IVC—longitudinal view	>0.48	0.67 [0.55–0.78]	0.69 [0.55–0.82]	0.63 [0.47–0.78]	0.69 [0.55–0.82]	0.63 [0.47–0.78]	1.90 [1.22–2.96]	0.48 [0.30–0.78]
IVC—transversal view	>0.58	0.68 [0.57–0.80]	0.54 [0.39–0.69]	0.86 [0.70–0.95]	0.83 [0.65–0.94]	0.59 [0.44–0.72]	3.80 [1.62–8.93]	0.53 [0.38–0.75]
NT-proBNP	>340 ng/L	0.76 [0.65–0.87]	0.77 [0.62–0.89]	0.74 [0.57–0.88]	0.79 [0.64–0.90]	0.72 [0.55–0.86]	3.01 [1.67–5.40]	0.31 [0.17–0.55]
Lower limb edema	Presence	-	0.39 [0.26–0.53]	0.87 [0.74 –0.95]	0.78 [0.58–0.91]	0.55 [0.43–0.67]	3.05 [1.34–6.91]	0.70 [0.55–0.89]
Jugular distension	Presence	-	0.22 [0.12–0.36]	0.96 [0.85–0.99]	0.86 [0.57–0.98]	0.52 [0.41–0.63]	5.22 [1.23–22.15]	0.81 [0.70–0.95]
Hepato-jugular reflux	Presence	-	0.41 [0.28–0.55]	0.91 [0.80–0.98]	0.85 [0.65–0.96]	0.57 [0.45–0.69]	4.79 [1.78–12.90]	0.65 [0.51–0.82]
Ascite	Presence	-	0.06 [ 0.01–0.15]	1.00 [0.92–1.00]	1.00 [0.29–1.00]	0.48 [0.38–0.58]	Inf	0.94 [0.89–1.01]
**To detect an RAP > 14 mmHg**								
RVSI	>0.182	0.69 [0.48–0.90]	0.71 [0.29–0.96]	0.77 [0.65–0.86]	0.25 [0.09–0.49]	0.96 [0.87–1.00]	3.10 [1.62–5.90]	0.37 [0.11–1.21]
FVSI	>0.45	0.93 [0.87–0.98]	0.82 [0.48–0.98]	0.93 [0.85–0.97]	0.60 [0.32–0.84]	0.98 [0.91–1.00]	11.73 [5.16–26.6]	0.20 [0.06–0.69]
IVC—longitudinal view	<0.39	0.75 [0.61–0.88]	0.67 [0.30–0.93]	0.62 [0.50–0.72]	0.16 [0.06–0.32]	0.94 [0.84–0.99]	1.74 [1.02–2.98]	0.54 [0.21–1.38]
IVC—transversal view	<0.47	0.81 [0.64–0.99]	0.89 [0.52–1.00]	0.81 [0.70–0.89]	0.36 [0.17–0.59]	0.98 [0.91–1.00]	4.57 [2.71–7.72]	0.14 [0.02–0.88]
NT-proBNP	>359 ng/L	0.61 [0.43–0.79]	0.82 [0.48–0.98]	0.56 [0.43–0.68]	0.23 [0.11–0.39]	0.95 [0.83–0.99]	1.85 [1.26–2.73]	0.33 [0.09–1.16]
Lower limb edema	Presence	-	0.55 [0.23–0.83]	0.77 [0.67–0.85]	0.22 [0.09–0.42]	0.93 [0.85–0.98]	2.34 [1.21–4.51]	0.59 [0.31–1.14]
Jugular distension	Presence	-	0.27 [0.06–0.61]	0.88 [0.79–0.94]	0.21 [0.05–0.51]	0.91 [0.83–0.96]	2.23 [0.73–6.79]	0.83 [0.57–1.20]
Hepato-jugular reflux	Presence	-	0.64 [0.31–0.89]	0.79 [0.69–0.87]	0.27 [0.12–0.48]	0.95 [0.87–0.99]	3.01 [1.66–5.49]	0.46 [0.21–1.01]
Ascite	Presence	-	NA *	0.97 [0.91–0.99]	NA *	0.89 [0.81–0.94]	NA *	1.03 [1.00–1.07]

* Cannot be calculated due to a lack of true positives. Values are expressed as values with [95% confidence interval]. AUC: Area under ROC curve. FVSI: Femoral venous stasis index. IVC: Inferior vena cava. NA: Not applicable. NLR: Negative likelihood ratio. NPV: Negative predictive value. NT-pro-BNP: N-terminal pro-brain natriuretic pressure. PLR: Positive likelihood ratio. PPV: Positive predictive value. RAP: Right atrial pressure. RVSI: Renal venous stasis index.

## Data Availability

The raw data supporting the conclusions of this article will be made available by the authors on request.

## Abbreviations

The following abbreviations are used in this manuscript:

ANOVA	Analysis of Variance
ARHF	Acute Right Heart Failure
DUS	Doppler ultrasound
ECG	Electrocardiogram
FVSI	Femoral Venous Stasis Index
IVC	Inferior Vena Cava
MHz	Megahertz
NT-proBNP	N-terminal pro-B-type Natriuretic Peptide
NPRE	Natriuretic Response Prediction Equation
PH	Pulmonary Hypertension
RA	Right Atrium
RAP	Right Atrial Pressure
RHC	Right Heart Catheterization
ROC	Receiver Operating Characteristic
RVSI	Renal Venous Stasis Index
